# Comprehensive Analysis of Clinicopathological and Molecular Features to Predict Anti-PD-1-Based Therapy Efficacy in Patients with Advanced Gastric Signet Ring Cell Carcinoma

**DOI:** 10.3390/jpm13010115

**Published:** 2023-01-04

**Authors:** Yan Hu, Nuo Chen, Ren-Ze Huang, Dong-Liang Chen

**Affiliations:** Collaborative Innovation Center for Cancer Medicine, Sun Yat-sen University Cancer Center, State Key Laboratory of Oncology in South China, Sun Yat-sen University, Guangzhou 510060, China

**Keywords:** PD-1 inhibitor, gastric cancer, gastric signet-ring cell carcinoma, predictive factors, efficacy

## Abstract

Background: Signet ring cell carcinoma (SRCC) is a specific type of gastric cancer. The clinicopathological and molecular characteristics that can be used to predict the response to anti-PD-1 therapy for these patients are still not clear. Methods: Patients with advanced SRCC who received first-line anti-PD-1-based treatment were enrolled in this study. The clinicopathological characteristics of these patients were obtained from their medical records. The molecular features of these patients were analyzed by means of a next-generation-sequencing-based panel. The predictive significance of clinicopathological and molecular features for efficacy was analyzed. Results: A total of 71 patients with measurable lesions were included in this study, among which 46 patients had enough tissues for next-generation sequencing. The overall objective response rate (ORR) was 46.4%. ORR was significantly higher in mismatch repair (MMR)-deficient (dMMR) patients than in MMR-proficient (pMMR) patients, in patients with lymph node metastasis only than those with other metastasis sites, and in patients with an Eastern Cooperative Oncology Group performance status (ECOG PS) of 0 than with a PS of 1 or 2. The progression-free survival was significantly longer in patients with dMMR, lymph node metastasis only, PD-L1 combined positive score (CPS) ≥ 5, and CDH1 wild type. Conclusions: Several clinicopathological and molecular features are associated with anti-PD-1 treatment efficacy in SRCC, which might be used to identify patients who can benefit most from these therapies.

## 1. Introduction

Gastric cancer is one of the most commonly diagnosed malignancies and is a leading cause of cancer-associated death worldwide, accounting for an estimated 989,600 new cases and 738,000 deaths every year [1]. Among these, approximately 40% of gastric cancer cases occur in China, and most of them are diagnosed at an advanced stage. Traditionally, the main treatment for these patients is chemotherapy, and the median overall survival for advanced gastric cancer patients is only about one year [2]. So far, only trastuzumab and ramucirumab have been approved for targeted therapy in advanced gastric cancer [3]. However, only about 15–20% of HER-2 positive patients can benefit from trastuzumab [3], while a combination of ramucirumab and chemotherapy increased progression-free survival but not overall survival [4]. Signet ring cell carcinoma (SRCC) is a specific type of gastric cancer, defined as gastric cancer consisting of at least 50% signet ring cells in the pathologic specimen according to the World Health Organization (WHO) classification, accounting for 8–30% of gastric cancer patients [5,6]. Studies have shown that advanced SRCC has a distinct epidemiology and molecular features—for example, SRCC is more often observed in younger women, is more invasive and has worse a prognosis, and is less sensitive to chemotherapy than non-SRCC [7,8]. Moreover, SRCC is not particularly affected by targeted therapy due to the low expression of HER-2 in these patients [9]. Therefore, it is essential to explore the molecular characteristics and find new therapeutic strategies for SRCC.

In recent years, it has been found that the blockade of immune checkpoint molecules with monoclonal antibodies is a successful therapy in several tumors [10]. In the tumor microenvironment, programmed death ligand 1 (PD-L1), which is a B7 family ligand, can bind to its receptor programmed death-1 (PD-1) to influence tumor-specific T cells, induce apoptosis, and inhibit the activity of CD8+ T cells, leading to immune evasion in tumors [11]. Accordingly, the blockade of this interaction with immune checkpoint inhibitors can restore the antitumor function of T cells [12]. An increasing number of clinical trials have shown that anti-PD-1/PD-L1 monoclonal antibodies are efficient immunotherapy approaches in different cancers [13,14]. 

For gastric cancer, the ATTRACTION-2 trial confirmed the efficacy of nivolumab, a human IgG4 monoclonal antibody, against PD-1 in patients with advanced gastric cancer after two or more lines of chemotherapy, regardless of PD-L1 expression [15]. On the basis of this, nivolumab has been approved for advanced gastric cancer patients in Japan. Another PD-1 antibody, pembrolizumab, also showed encouraging anti-tumor activity and acceptable safety in advanced gastric cancer [16]. These trails confirmed the efficacy of anti-PD-1 therapy for all types of gastric cancer, including SRCC. Considering the predictive biomarkers, phase II and III trails have indicated that PD-L1 expression evaluated as a combined positive score (CPS) in both tumor cells and immune cells is associated with efficacy—a higher CPS is correlated with greater treatment effect [17]. In addition, high microsatellite instability or mismatch repair (MMR) deficiency has been demonstrated to be a predictor of the response to PD-1 antibodies in solid tumors, including gastric cancer [18]. In addition, some studies showed that tumor mutation burden (TMB) and EBV positivity were also correlated with the response to PD-1 antibodies [19]. However, the predictive factors of anti-PD-1 response in SRCC have not been determined. 

In this study, in order to identify the predictive factors that are associated with the therapeutic efficacy of anti-PD-1 on SRCC, we analyzed the association between clinicopathological and molecular characteristics and the objective response rate (ORR) and progression-free survival (PFS) in advanced SRCC patients receiving anti-PD-1-based therapy. 

## 2. Patients and Methods

### 2.1. Patients

A real-world study (no. NCT04086888) was performed to evaluate the efficacy of anti-PD-1 therapy in patients with advanced gastric cancer from August 2018 to March 2022 at Sun Yat-sen University Cancer Center. In the present study, we evaluated the efficacy and predictive factors of anti-PD-1 therapy in SRCC patients. The inclusion criteria were as follows: (1) pathologically confirmed SRCC; (2) initial stage IV; (3) with measurable lesions for efficacy evaluation; (4) adequate bone marrow, hepatic, and renal function; (5) Eastern Cooperative Oncology Group performance status (ECOG PS) of 0–2; and (6) the patient received at least one treatment with anti-PD-1-based therapy. All of the patients signed written informed consent before receiving anti-PD-1 treatment. The study was approved by the ethics committee of the Sun Yat-sen University Cancer Center. All of the patients were followed up regularly until death or last contact. 

### 2.2. Molecular Characteristics

Molecular characteristics were analyzed by using formalin-fixed paraffin-embedded (FFPE) tissues. HER-2 status was determined by immunohistochemistry (IHC) using an anti-HER-2 monoclonal antibody and fluorescence in situ hybridization (FISH) using the PathVysion HER-2 probe kit. HER-2 positivity was defined as IHC 3+ or IHC 2+ and FISH positivity. PD-L1 expression was analyzed by means of IHC using an anti-PD-L1 mouse monoclonal antibody (clone 22C3, Dako: Cat No.M3653), and PD-L1 positivity was defined as a presence of ≥1% in tumor cells (TC) and immune cells (IC) with membranes. CPS was defined as the sum of all PD-L1+ cells (tumor cells, lymphocytes, and macrophages) divided by the total number of viable tumor cells. MMR status was routinely assessed by IHC staining of four proteins (MLH1, MSH2, MSH6, and PSM2). Tumors with at least one lost protein were considered as MMR deficient (dMMR), whereas tumors that showed intact MMR protein expression were considered as MMR proficient (pMMR). EBV status was evaluated by chromogenic in situ hybridization for EBV-encoded RNA (EBER) using the fluorescein-labeled oligonucleotide probes (INFORMEBER Probe; Ventana) as previously described. Genomic alterations were determined by performing DNA sequencing using a WESPlus gene panel (an upgraded version of the standard whole-exome sequencing (WES), HaploX Biotechnology) with FFPE tissue samples as previously described [20], able to detect the gene mutations, copy number variants, and deletions, as well as fusion genes. TMB was defined as the number of somatic, non-synonymous, and indel mutations per megabase (mt/Mb) of the genome examined. Known germline alterations found in the dbSNP database were not counted. High TMB was defined as more than 10 mutations per megabase. 

### 2.3. Assessment and Statistical Analysis

The response rate was assessed on the basis of the response evaluation criteria in the Solid Tumors version 1.1 (RECIST1.1) guidelines. The tumor response included complete response (CR), partial response (PR), stable disease (SD), and progressive disease (PD). The objective response rate (ORR) was defined as the percentage of patients who received CR and PR, and the disease control rate (DCR) was defined as the percentage of the patients who received CR, PR, and SD. Progression-free survival (PFS) was calculated from the start of PD-1 antibody treatment to the date of progression or death. Overall survival (OS) was calculated from the start of PD-1 antibody treatment to the date of death or last contact. 

Statistical analyses were performed with SPSS 17.0 software (SPSS Inc., Chicago, IL, USA). The comparison of ORR and DCR between different clinical and pathological characteristics was performed by means of a chi-squared test or Fisher’s exact test. PFS was evaluated by means of the Kaplan–Meier method with the log-rank test. Multivariate analysis for PFS was performed on clinicopathological and molecular characteristics that had a significant impact on PFS. All of the tests were two sided, and a *p*-value of <0.05 was considered statistically significant. 

## 3. Results

### 3.1. Patients’ Characteristics

A total of 71 SRCC patients were enrolled in this study. The baseline characteristics of these patients are shown in Table S1. The median age was 52 years (range, 29–78 years), and 47 (66.1%) of the patients were male. Twenty-one (29.5%) of the patients presented a PS of 0, and the remaining patients presented a PS of 1 or 2 at the beginning of anti-PD-1 therapy. Most (90.1%) of the patients had diffused-type tumors, and the remainder (9.9%) had mixed-type tumors. Thirty-seven (52.1%) of the patients had lymph node metastasis, 38.0% of the patients had peritoneal metastasis, and 21.1% had liver metastasis. Four (5.6%) of the patients had HER-2-positive tumors, and four of the patients presented with dMMR. Five of the patients were EBV positive. Thirty-one (43.6%) of the patients had PD-L1 expression of CPS ≥ 5. 

### 3.2. Molecular Alteration in Primary and Metastatic Tissues

**Figure 1:**
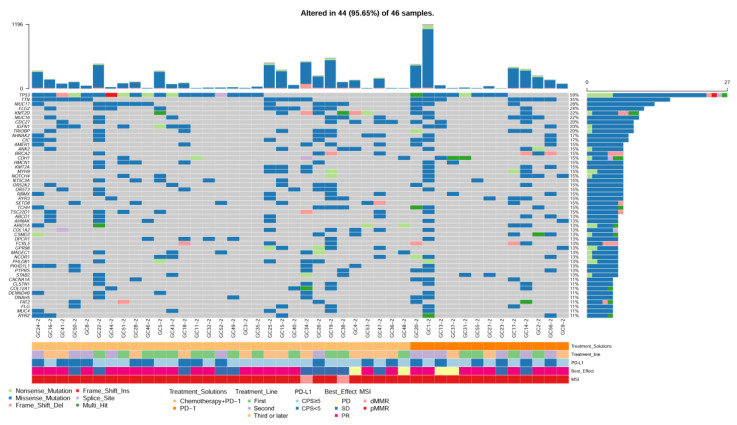
Genomic landscape of gastric Signet ring cell carcinoma (SRCC) The most frequently mutated genes of 46 primary tumor tissues.

Forty-six primary tumor tissues and fourteen metastatic tissues were sequenced with a WESPlus gene panel (HaploX Biotechnology, Shenzhen, China). Overall, 311 mutations spanning 135 genes from the primary tumor tissues and 297 mutations spanning the 128 genes form the metastatic tumor tissues were identified. In addition, 158 common mutations from 74 genes were detected in the paired primary tumor tissues and metastatic tumor tissues. The most commonly identified gene alterations in primary tumor tissues are listed in Figure 1. The most frequently altered genes were TP53 (59%), TTN (48%), OBSCN (39%), HMCN2 (37%), and TRPV1 (37%). In addition, we compared the gene alteration in 14 paired primary and metastatic tissues (Figure S1). There was no significant difference in gene mutations between the primary and metastatic tumor tissues (McNemar’s test, *p* > 0.05). The median TMB was 8.7 Muts/Mb in our cohort, and the TMB was slightly higher in metastatic tumors than in primary tumors, although not statistically significant (Figure S2). The copy number variant was also detected in the paired primary and metastatic tumor tissues (Figure S3). 

### 3.3. Clinicopathological and Molecular Characteristics Associated with Anti-PD-1 Response in SRCC

Of the 71 patients, 0 (0%) received CR, 33 (46.4%) received PR, 21 (29.5%) received SD, and 17 (24.1%) received PD, resulting in an ORR of 46.4% and a DCR of 75.9%. A significantly higher ORR was observed in patients with a PS of 0 (76.1%) than patients with a PS of 1 or 2 (34.0%) (*p* = 0.001). ORR was significantly higher in patients with lymph node metastasis only (77.7%) than patients with other metastatic sites (35.8%) (*p* = 0.002). However, no other clinicopathological factors were associated with ORR (Table 1). As for molecular characteristics, ORR was significantly higher in patients with dMMR (100.0%) than in patients with pMMR (43.2%) (*p* = 0.027). ORR tended to be higher in patients with PD-L1 CPS ≥ 5 (58.0%) than in patients with PD-L1 CPS < 5 (37.5%) (*p* = 0.085). There were no other molecular factors associated with ORR in SRCC patients (Table 2). 

### 3.4. Clinicopathological and Molecular Factors to Predict PFS

**Figure 2:**
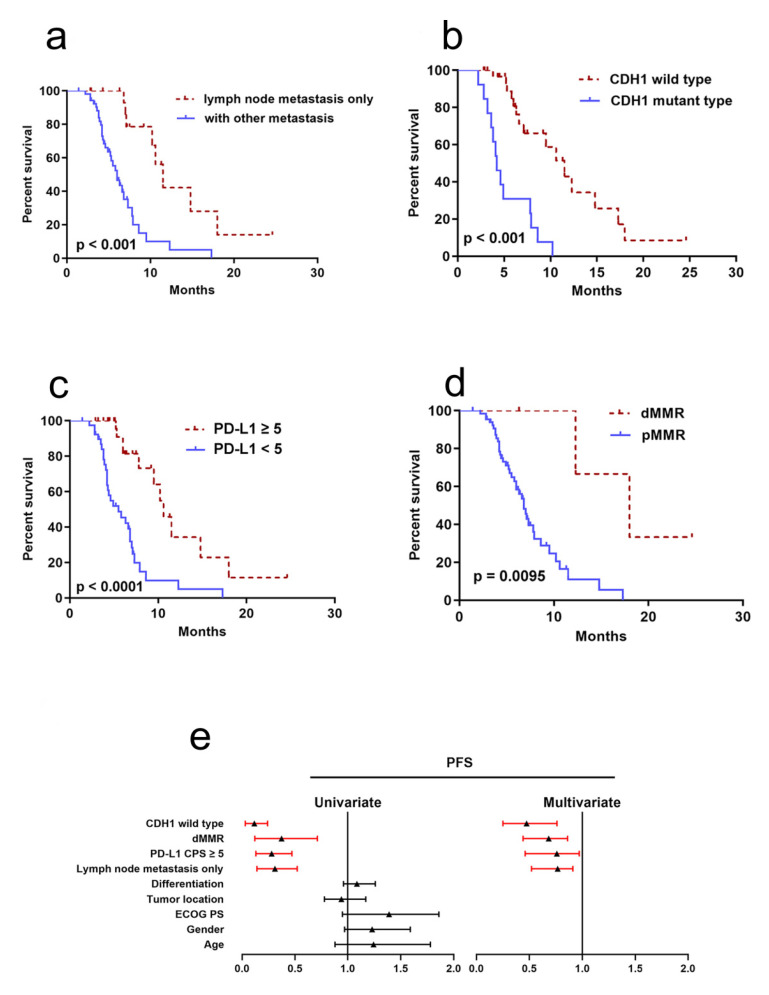
Clinicopathological and molecular factors to predict PFS. (**a**) Kaplan–Meier curve of lymph node metastasis (*p* < 0.001); (**b**) Kaplan–Meier curve of CDH1 (*p* < 0.001); (**c**) Kaplan–Meier curve of PD-L1 (*p* < 0.001); (**d**) Kaplan–Meier curve of MMR (*p* = 0.0095); (**e**) forest plot of univariate or multivariable Cox proportional hazard regression indicating the impact of different characteristics on PFS.

For the 71 patients, the median PFS for anti-PD-1 therapy was 5.3 (95% CI 3.3–9.6) months with a median follow-up period of 12.3 months (range 1.4–24.6 months). The PFS was significantly longer in patients with lymph node metastasis only (median 11.5 months) than in those with other metastatic sites (median 6.0 months) (*p* = 0.00003) (Figure 2a), in CDH1 wild type (median 11.5 months) than in CDH1 mutant type (median 4.2 months) (*p* < 0.001) (Figure 2b), in PD-L1 CPS ≥ 5 (median 10.6 months) than in PD-L1 < 5 (5.5 months) (*p* < 0.001) (Figure 2c), and in patients with dMMR (median 18.0 months) than in patients with pMMR (median 6.8 months) (*p* = 0.04) (Figure 2d). Univariate analysis showed that dMMR, lymph node metastasis only, PD-L1 CPS CPS ≥ 5, and CDH1 wild type were significantly associated with longer PFS. Multivariate analysis showed that dMMR, PD-L1 CPS CPS ≥ 5, CDH1 wild type, and lymph node metastasis only were independent prognostic factors for PFS (Figure 2e and Table S2). 

## 4. Discussion

Gastric SRCC has different clinicopathological features, with poor tissue differentiation, greater invasiveness, and poor prognosis, especially in advanced-stage patients [21,22]. Gastric SRCC belongs to the diffuse type and has been reported to have a low mutation rate, high frequency of TP53 alteration [23,24], foci deletion of FHIT, and amplification of multiple genes, including FGFR2, CD44, CCNE1, and so on [25]. A recent study reported that the CLDN18-ARHGAP26/6 fusion gene was frequently identified in gastric SRCC patients, and patients with this fusion gene had worse survival outcomes [26]. In the present study, we found that the TP53 mutation was frequently observed in advanced gastric SRCC, which is in line with previous reports. We found that the mutation of TTN, OBSCN, and HMCN2 was also common in these patients. The TMB was slightly higher than that of non-SRCC patients. More importantly, we reported for the first time that there is no significant difference between the genomic profiles of the primary and metastatic tissues.

Anti-PD-1 therapy has been confirmed to be effective in advanced gastric cancer patients. Our group also confirmed the clinical benefit of PD-1 antibody therapy in gastric cancer patients [27]. However, most gastric cancer patients are resistant to anti-PD-1 therapy, and only some patients benefit from this treatment [19]. The predictive biomarkers that can be used to predict anti-PD-1 effect are controversial. For instance, PD-L1 has been proven to be a predictive biomarker of anti-PD-1 efficacy in several tumor types. However, the predictive role of PD-L1 expression in gastric cancer is controversial [28]. KENOTE-061 and KENOTE-062 trials showed better survival in patients with PD-L1-positive tumors after pembrolizumab treatment [17,29]. On the other hand, data from Checkmate032, JAVELIN Gastric 300, and ATTRCTION-2 trials did not support the concept of PD-L1 positivity as a predictive biomarker of anti-PD-1 efficacy [15,30,31]. Moreover, most of the clinical trials included all types of patients, and the effect and PD-1 antibody in gastric SRCC specifically and its predictive factors are still unknown. In the present study, we confirmed that anti-PD-1 therapy is effective in gastric SRCC patients. Furthermore, a PS of 0 and dMMR were associated with higher ORR and longer survival time in gastric SRCC treated with PD-1 antibody, and this is in line with a previous study that was conducted in common gastric cancer [19,32]. 

High TMB has been found to be associated with the ORR of anti-PD-1 therapy for gastric cancer [19]. In this study, we performed whole-exon sequencing and calculated the TMB in SRCC, which is relatively higher than in common gastric cancer. A high TMB correlated with higher ORR, but this was not statistically significant. One of the reasons for this might be that most of the patients with high TMB had a dMMR status. Thus, whether TMB can be used as an independent predictor of anti-PD-1 therapy requires further investigation. Previous studies have indicated that EBV positivity in the tumor is associated with a response to the PD-1 antibody [33]. However, in the present study, EBV status was not associated with ORR and PFS in SRCC. 

Interestingly, we identified that lymph node metastasis only and CDH1 wild type were associated with higher ORR and longer PFS in gastric SRCC treated with PD-1 antibody. Previously, a study found that lymphovascular invasion is positively associated with TMB and PD-L1 expression in resected lung cancer [34], and a link was found between lymph node metastasis and higher ORR of anti-PD-1 response in gastric cancer, although this was not statistically significant [32]. Our study revealed for the first time that lymph node metastasis is only associated with high ORR and longer PFS of gastric SRCC treated with anti-PD-1 therapy. However, further clinical trials with larger patient cohorts are needed to confirm this. Previous studies reported that a higher mutation rate of CDH1 was observed in SRCC patients [35,36], which is in accordance with our results. In this study, we found that CDH1 mutation is associated with lower ORR and adverse PFS in SRCC; to our knowledge, our study revealed for the first time the predictive role of CDH1 mutation for anti-PD-1 efficacy and prognosis in SRCC. However, further studies with larger cohorts of patients are needed to confirm this. 

The predictive of ECOG PS for anti-PD-1 response is still controversial. For instance, a recent meta-analysis showed that there was no significant association between ECOG PS and anti-PD-1 response in advanced solid tumors [37], whereas another study demonstrated that the ECOG score was significantly associated with survival in NSCLC treated with PD-1/PD-L1 blockade [38]. However, further studies are needed to confirm this in larger cohorts of patients. 

There are some limitations in this study. First, this was a single-center study with a limited sample size. Second, due to the lack of tissues, not all of the patients underwent gene sequencing analysis. Third, this was a single-arm study without a control. 

In conclusion, we identified some clinicopathological and molecular characteristics that are associated with the efficacy and PFS of gastric SRCC receiving anti-PD-1-based treatment. The combination of these features might help to select the patients who can really benefit from anti-PD-1 therapy. However, further studies with larger cohorts are needed to confirm precise biomarkers for anti-PD-1′s efficacy in gastric SRCC. 

## Figures and Tables

**Table 1:** Clinicopathological characteristics associated with response to anti-PD-1 therapy in SRCC.

N = 71		All	Responder	Non-Responder	ORR	*p*-Value
Age	<65	34	15	19	44.1%	0.702
	≥65	37	18	19	48.6%	
Gender	Male	47	20	27	42.5%	0.353
	Female	24	13	11	54.1%	
ECOG PS	0	21	16	5	76.1%	0.001
	≥1	50	17	33	34.0%	
Primary tumor location	EGJ/Cardia	11	4	7	36.3%	0.622
	Body	32	17	15	53.1%	
	Antrum	24	11	13	45.8%	
	Unknown	4	1	3	25.0%	
Differentiation	Moderately	2	1	1	50.0%	0.674
	Poorly	65	31	34	48.4%	
	Unknown	4	1	3	25.0%	
Lauren classification	Intestinal	0	0	0	-	0.317
	Diffuse	64	31	33	48.4%	
	Mixed	7	2	5	28.5%	
Previous gastrectomy	Yes	8	3	5	37.5%	0.589
	No	63	30	33	47.6%	
Lymph node metastasis only	Yes	18	14	4	77.7%	0.002
	No	53	19	34	35.8%	

Abbreviations: ECOG, Eastern Cooperative Oncology Group; EGJ, esophagogastric junction; ORR, objective response rate.

**Table 2:** Molecular features associated with response to anti-PD-1 therapy in SRCC.

Characteristics		Detected	Responder	Non-Responder	ORR	*p*-Value
PD-L1 CPS	≥5	40	15	25	37.5%	0.085
	<5	31	18	13	58.0%	
HER-2+	Yes	5	4	1	80.0%	0.119
	No	66	29	37	43.9%	
dMMR	Yes	4	4	0	100.0%	0.027
	No	67	29	38	43.2%	
EBV+	Yes	4	3	1	75.0%	0.197
	No	55	23	32	41.8%	
TMB	≥10	9	6	3	66.6%	0.494
	<10	37	20	17	54.0%	
TP53 mutation	Yes	24	13	11	54.1%	0.736
	No	22	13	9	59.0%	
PIK3CA mutation	Yes	7	3	4	42.8%	0.428
	No	39	23	16	58.9%	
CDH1 mutation	Yes	13	6	7	46.1%	0.373
	No	33	20	13	60.6%	
MET mutation	Yes	3	1	2	33.3%	0.402
	No	43	25	18	58.1%	
KRAS mutation	Yes	4	2	2	50.0%	0.783
	No	42	24	18	57.1%	
FGFR amplification	Yes	2	1	1	50.0%	0.849
	No	44	25	19	56.8%	
MYC amplification	Yes	5	3	2	60.0%	0.868
	No	41	23	18	56.0%	
ERBB2 amplification	Yes	4	1	3	25.0%	0.183
	No	42	25	17	59.5%	

Abbreviations: EBV, Epstein–Barr virus; MMR, mismatch repair; dMMR, deficient mismatch repair; pMMR, proficient mismatch repair; PD-L1, programmed cell death ligand 1.

## Data Availability

All the data are provided in the article and the supplementary data.

## Supplementary Materials

The following supporting information can be downloaded at: https://www.mdpi.com/article/10.3390/jpm13010115/s1, Figure S1: Comparison of gene mutations between 14 paired primary and metastatic tissues using McNemar’s test (*p* > 0.05); Figure S2: Comparison of median TMB between primary and metastatic tissues (Wilcox test, *p* = 0.068); Figure S3: Copy number variations detected in the paired primary and metastatic tumor tissues; Table S1: Patient’s baseline clinicopathological characteristics; Table S2: Univariate and multivariate analyses of predictors for progression-free survival in SRCC patients.

## Abbreviations

SRCC: signet ring cell carcinoma; WHO: The World Health Organization; PD-L1: programmed death ligand 1; PD-1: programmed death-1; CPS: combined positive score; MMR: mismatch repair; TMB: tumor mutation burden; ORR: overall objective response rate; PFS: progression-free survival; ECOG PS: Eastern Cooperative Oncology Group Performance Status; FFPE: formalin-fixed paraffin-embedded; IHC: immunohistochemistry; FISH: fluorescence in situ hybridization; TC: tumor cells; IC: immune cells; dMMR: MMR-deficient; pMMR: MMR-proficient; EBER: EBV-encoded RNA; WES: whole-exome sequencing; CR: complete response; PR: partial response; SD: stable disease; PD: progressive disease; DCR: disease control rate; OS: overall survival.
