# Supplementary material for: Comprehensive Analysis of Clinicopathological and Molecular Features to Predict Anti-PD-1-Based Therapy Efficacy in Patients with Advanced Gastric Signet Ring Cell Carcinoma
Source: J Pers Med. 2023 Jan 4;13(1):115. doi: 10.3390/jpm13010115 (PMC9861489; doi:10.3390/jpm13010115)
Supplement: Supplementary file 1 [file jpm-13-00115-s001.zip › jpm-2128623-supplementary.pdf]

Table S1. Patient's baseline clinicopathological characteristics

**Characteristics**		**Number**
Age	Median (Range)	52 (29-78)
Gender	Male	47 (66.1%)
	Female	24 (33.9%)
Race	Asian	71 (100%)
ECOG performance status	0	21 (29.5%)
	1	32 (45.0%)
	2	18 (25.5%)
Primary tumor location	EGJ/Cardia	13 (18.3%)
	Body	32 (45.0%)
	Antrum	22 (30.9%)
	Unknown	4 (5.8%)
Histological grade	Moderately differentiated	2 (2.8%)
	Poorly differentiated	65 (91.5%)
	Unknown	4 (5.4%)
Lauren Classification	Intestinal	0 (0.0%)
	Diffuse	64 (90.1%)
	Mixed	7 (9.9%)
Metastatic sites	Lymph nodes	37 (52.1%)
	Peritoneum	27 (38.0%)
	Liver	15 (21.1%)
	Bone	5 (7.0%)
	Others	12 (16.9%)
Previous gastrectomy	Yes	8 (11.2%)
	No	63 (88.8%)
HER-2 status	Positive	4 (5.6%)
	Negative	67 (94.4%)
EBV in situ hybridization	Positive	5 (7.0%)
	Negative	64 (90.1%)
	Unknown	2 (2.9%)
MSI/MMR testing	MSI-H/dMMR	4 (5.6%)
	MSI-L/MSS/pMMR	67 (94.4%)
PD-L1 status	CPS≥5	31 (43.6%)
	CPS<5	27 (56.4%)

Abbreviations: ECOG, Eastern Cooperative Oncology Group; EGJ, esophagogastric junction; EBV, Epstein-Barr virus; MSI, microsatellite instability; MMR, mismatch repair; MSI-H, high-level microsatellite instability; MSI-L, low-level microsatellite instability; dMMR, deficient mismatch repair; pMMR, proficient mismatch repair; PD-L1,programmed cell death ligand 1.

Table S2. Univariate and multivariate analyses of predictors for progression-free survival in SRCC patients

Characteristics	Univariate analysis		Multivariate analysis	
	HR[b](95%CI[c])	*P*	HR[b](95%CI[c])	*P*
Age	1.07(0.88-1.78)	0.236	-	-
Gender	1.13(0.97-1.59)	0.356	-	-
ECOG PS	1.36(0.95-1.86)	0.086	-	-
Tumor location	0.87(0.78-1.17)	0.426	-	-
Differentiation	1.04(0.96-1.26)	0.247	-	-
Lymph node metastasis only	0.27(0.14-0.52)	<0.001[a]	0.87(0.52-0.91)	0.035[a]
PD-L1 CPS ≥ 5	0.24(0.13-0.47)	<0.001[a]	0.85(0.46-0.97)	0.041[a]
dMMR	0.29(0.12-0.71)	0.0095[a]	0.75(0.44-0.86)	0.021[a]
CDH1 wild type	0.08(0.03-0.24)	<0.001[a]	0.41(0.25-0.76)	0.019[a]

[a]$P < 0.05$.

[b]HR, hazard ratio.

[c]CI, confidence interval.

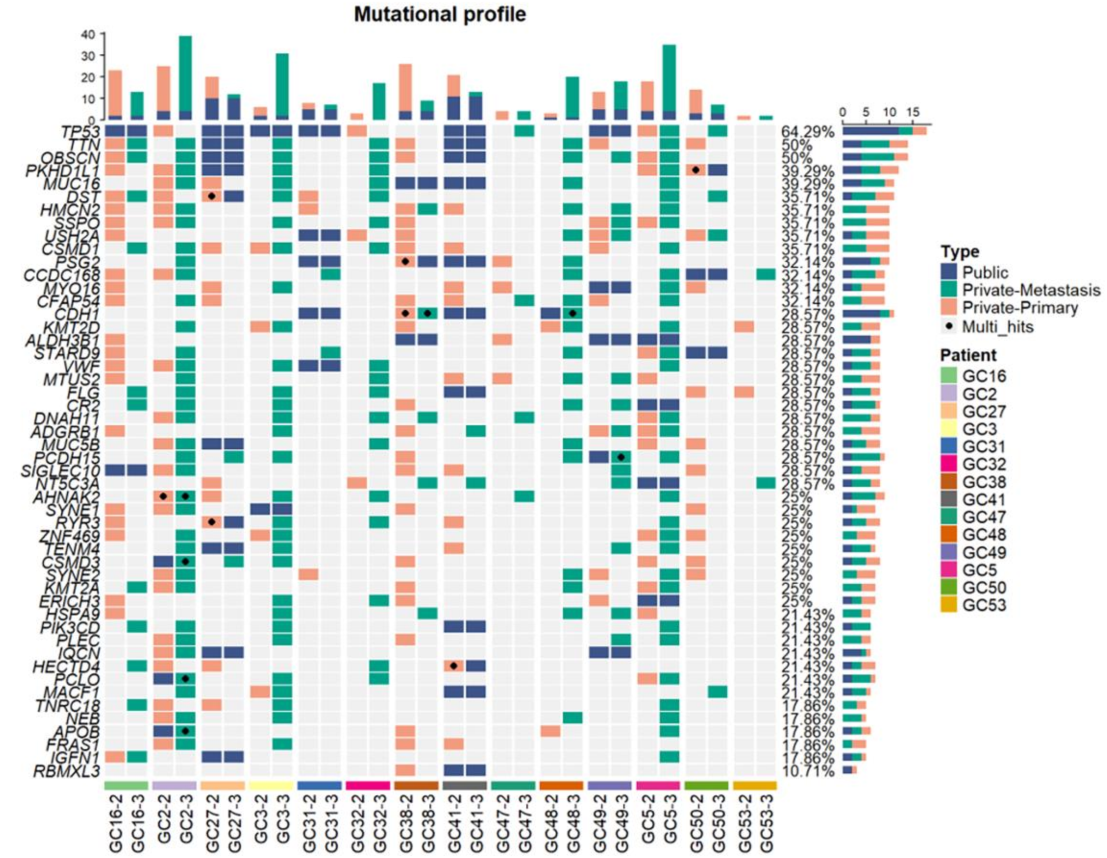

Figure S1. Comparison of gene mutations between14 paired primary and metastatic tissues using McNemar’s test (P > 0.05).

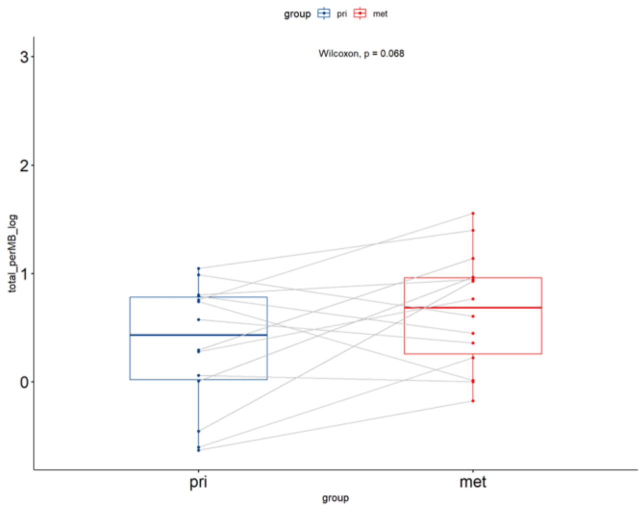

Figure S2. Comparison of median TMB between primary and metastatic tissues (Wilcox test, P = 0.068).

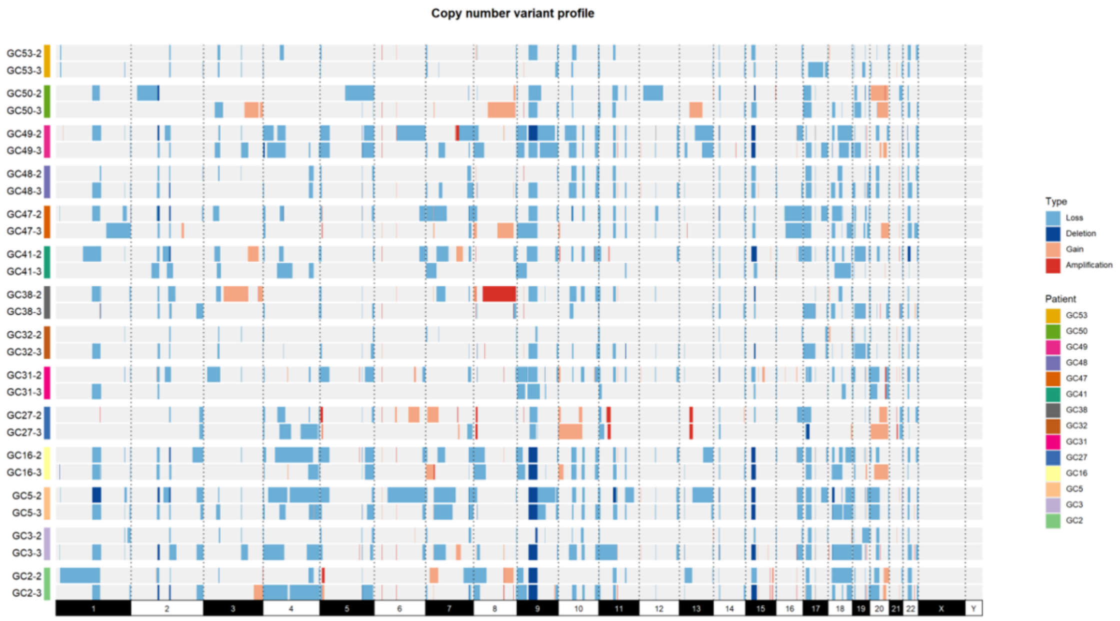

Figure S3. Copy number variations detected in the paired primary and metastatic tumor tissues.
